# Study on the Aging Mechanism of Boron Potassium Nitrate (BKNO_3_) for Sustainable Efficiency in Pyrotechnic Mechanical Devices

**DOI:** 10.1038/s41598-018-29412-8

**Published:** 2018-08-06

**Authors:** Junwoo Lee, Taewan Kim, Seung Un Ryu, Kyoungwon Choi, Gil Hwan Ahn, Jong Gyu Paik, Byungtae Ryu, Taiho Park, Yong Sun Won

**Affiliations:** 1 Chemical Engineering, Pohang University of Science and Technology (POSTECH), 77 Cheongam-Ro, Nam-gu, Pohang, Kyoungbuk Korea; 2 Hanwha Corporation Defense R&D Center, Daejeon, 34068 Korea; 3 Agency for Defense Development, Daejeon, 305-152 Korea; 4 Chemical Engineering, Pukyong National University, 365, Sinseon-Ro, Nam-gu, Busan, 48547 Busan, Korea

## Abstract

The aging of propellants in PMDs is considered to be one of the primary factors affecting the performance of PMDs. Thus, studies on the aging mechanism of propellants, which have not yet been addressed extensively, pose a solution to securing the sustainable operation of PMDs. We characterized one of the most commonly used commercial propellants (boron potassium nitrate (BKNO_3_)) and investigated its aging mechanism rigorously. Based on thermal analyses, we demonstrate that the decomposition of laminac, a polymer binder, is the fastest spontaneous reaction. However, it will not self-initiate at a storage temperature as high as 120 °C. The effect of the humidity level was examined by characterizing BKNO_3_ samples prepared. The heat of reaction and the reaction rate decreased by 18% and 67% over 16 weeks of aging, respectively. This is attributed to the oxide shells on the surface of boron particles. The formation of oxide shells could be confirmed using X-ray photoelectron spectroscopy and transmission electron microscopy–energy dispersive spectroscopy. In conclusion, surface oxide formation with the aging of BKNO_3_ will decrease its propulsive efficiency; oxidation reduces the potential energy of the system and the resulting oxide decreases the reaction rate.

## Introduction

Pyrotechnic mechanical devices (PMDs) that utilize pressure through explosions are a highly attractive topic in aerospace industries^1^. In such devices, zirconium potassium perchlorate (ZPP), boron potassium nitrate (BKNO_3_), and titanium hydride potassium perchlorate (THPP) are used as propellants (or pyro-initiators)^2–4^. These three types of propellants must have long-term stability to be utilized in practical applications^5^. That is, the reaction heat and reaction rate should be constant even after a long period of time. However, most PMDs are disposed periodically because of the aging phenomena of the propellants. Increasing the disposal period of explosives through research on the aging mechanism and controlling the aging can lead to a huge reduction in a national defense budget^6^.

Common PMDs comprise three components: an oxidizer, binder, and metal. The aging phenomena of explosives are considered to be caused by physical or chemical transitions through spontaneous reactions^7–11^ or by external factors such as humidity^12–16^. These phenomena can also be divided into thermodynamic and kinetic aspects. Thermodynamic aging means the reduction of the reaction heat in an explosion, resulting from a decrease in the energy level of reactants due to pre-chemical reactions among the components^17^ or due to external factors. On the other hand, kinetic aging leads to a decreased combustion rate and increased activation energy upon the formation of products because of side products and impurities. It can induce a reduction in the peak pressure of PMDs despite the constant heat of the reaction^18,19^.

**Figure 1:**
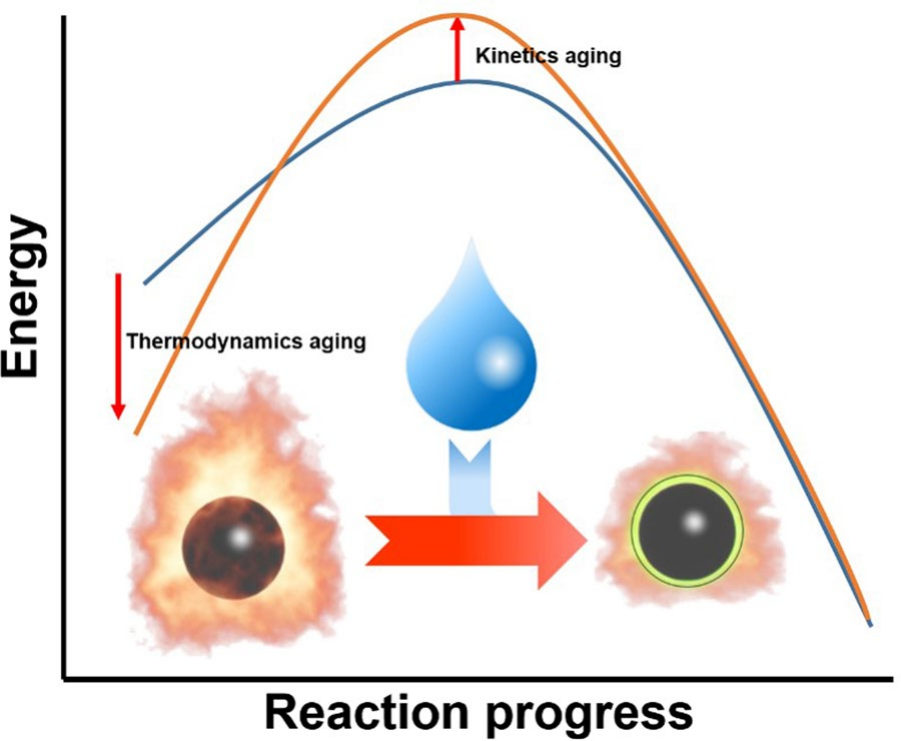
Schematic representation of aging mechanism of the propellant (BKNO_3_). The efficiency of the propellant is decreased by forming oxide shells on boron surfaces from humidity aging.

Here, we investigate the aging mechanism of BKNO_3_, which is well known to possess a higher peak pressure than that of the other two commonly used propellants (THPP and ZPP)^20–24^. Previous study on the aging of BKNO_3_ emphasized only pressure in PMD devices^6^. However, to clarify the mechanism, side chemical reaction of the propellant should be analyzed. BKNO_3_ comprises three components: boron, potassium nitrate, and laminac. Boron plays the role of an oxidization source in explosions^25^, whereas potassium nitrate (KNO_3_) donates oxygen to boron^26^. Laminac is a commercial polymer binder that causes boron and KNO_3_ to bind together closely^27^. A typical mixing ratio of these components is 23.7:70.7:5.6 on a weight percentage basis. In this system, boron reacts with KNO_3_, producing potassium metaborate (KBO_2_) exothermically. The resulting heat produces auto-acceleration, causes an explosion, and produces high pressure for the propulsion of PMDs^28^. Although boron is highly stable at high humidity levels and ambient temperatures, it is known to yield boronic acid (RB(OH)_2_) and boron oxide (B_2_O_3_) at above 300 °C^29^. Aging (mostly oxide formation) due to humidity should thus be considerable during the incorporation process into PMDs wherein the propellants are exposed to a very high humidity level to prevent an undesirable explosion (Fig. 1). However, aging due to external factors is less of an issue after this because BKNO_3_ is sealed in the PMDs. It is now necessary to consider internal factors for any further spontaneous aging for KNO_3_ and laminac. KNO_3_, a strong oxidizer, decomposes into KNO_2_ and oxygen at above 550 °C^26^. Therefore, the decomposition of KNO_3_ over an extended period can possibly induce undesirable pre-reactions with boron and decrease the energy level of the reactants. Moreover, the decomposition of laminac, which comprises polyester, results in micro phase separation. The reduced contact area between boron and KNO_3_ particles lowers the combustion rate and increases the activation energy^30^.

To investigate the aging of BKNO_3_ due to internal factors, we preferentially employed thermal analyses such as thermal gravimetric analysis (TGA) and differential scanning calorimetry (DSC)^31^. Spontaneous aging and its mechanisms were simulated using advanced kinetics and technology solutions (AKTS) with DSC data. The AKTS program is based on the Arrhenius–Sesták–Berggren theory that treats the reaction rate according to the activation energy, temperature, and reaction progress^32^. The equation is shown below,

1 $$\frac{d\alpha }{dt}=A\cdot \exp \,(-\frac{E}{RT}){(1-\alpha )}^{n}{\alpha }^{m},$$

where α is the reaction progress, *E* is the activation energy, and *A*, *n*, and *m* are reaction constants.

To investigate external factors, BKNO_3_ samples prepared under accelerated aging at 71 °C with a 50% relative humidity were characterized to measure the heats of reaction and the reaction rates using DSC profiles. X-ray photoelectron spectroscopy (XPS)^33^ and a transmission electron microscope (TEM) equipped with energy dispersive spectroscopy (EDS) were also used to confirm the oxidization of boron and identify oxide shells^34^ on the boron surface. The formation of surface oxide is supposed to decrease the potential energy (explosive energy) of the system and disturb the propagation of combustion^10^.

## Results

### Thermal properties of BKNO_3_

**Figure 2:**
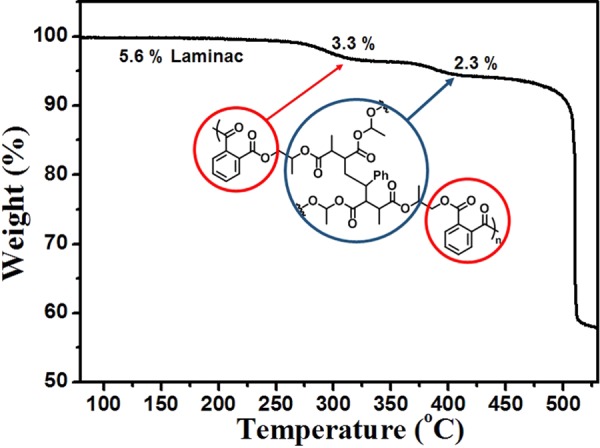
The TGA curve of BKNO_3_. The decomposition of laminac occurs from around 300 °C to 400 °C.

The TGA curve shown in Fig. 2 for BKNO_3_ in a nitrogen atmosphere indicates its thermal stability and the degradation of each component. There are three stages of weight loss in the TGA plot, indicating that the decomposition of the BKNO_3_ sample initiated at around 300, 410, and 500 °C. The first two stages are attributed to the weak stability of ester groups^35^ in laminac and the weight losses were 3.3% and 2.3%, respectively. The polymer binder was perfectly decomposed at 410 °C with a total weight loss of 5.6%. In the third stage, the oxidization of boron with KNO_3_ occurs, accompanied by a remarkable weight loss. The oxidization is the main reaction of the propellants, finally producing nitrogen dioxide gas^28,36^.

**Figure 3:**
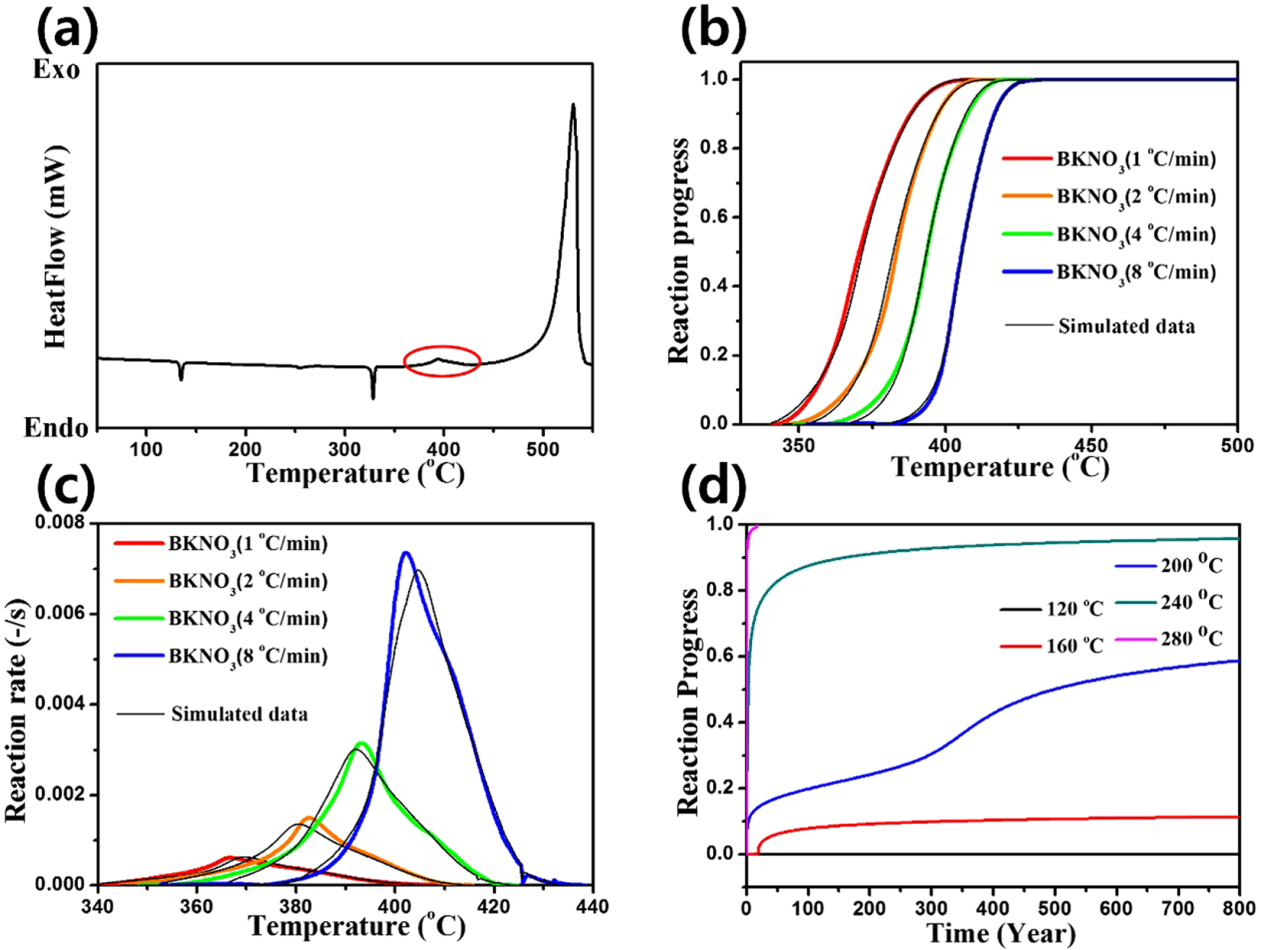
(**a**) The DSC profile of BKNO_3_. The first exothermic reaction is the decomposition of laminac (red circle) (**b**) Reaction progress vs. temperature and (**c**) reaction rate vs. temperature compared for various heating rates (1, 2, 4, and 8 °C/min). (**d**) Long-period reaction progress vs. time at various storage temperatures. Although the storage temperature is high (120 °C), the initiation of reaction (the decomposition of laminac) takes an infinite time.

In the DSC profiles shown in Fig. 3a, there are two types of exothermic reactions^37^. Ahead of the primary exothermic peak from boron oxidization at around 500 °C, a weak exothermic peak due to the decomposition of laminac was detected at 400 °C^38^. The exothermic decomposition of laminac at a relatively low temperature suggests that over an extended period, it is more likely to occur than primary boron oxidation. Therefore, the breakdown of laminac could affect the kinetic aging of the overall system by changing the morphology or internal structure of BKNO_3_^38,39^.

### Kinetic simulation for laminac decomposition

The simulated AKTS data are shown in Fig. 3b–d. AKTS was adopted to calculate the reaction progress and reaction rate according to the heating rate of the DSC data (Fig. 3b,c). It was also adopted to determine the long-term reaction progress related to the storage temperature (Fig. 3d) using the DSC data with varying heating rates (1, 2, 4, and 8 °C/min) (Supplementary Fig. S1). Here, the first exothermic reaction of laminac decomposition was studied to simulate its kinetic behavior. The resulting error factor of the simulation was about 0.08%, indicating an accurate estimation of the long-period reaction progress.

The reaction progress according to the heating rate is shown in Fig. 3b. The reaction progress α(*t*) of a DSC profile is expressed as

2 $$\alpha (t)=\frac{{\int }_{{t}_{o}}^{t}(S(t)-B(t))\,dt}{{\int }_{{t}_{o}}^{{t}_{end}}(S(t)-B(t))\,dt},$$

where *S*(*t*) is a DSC signal as a function of time with respect to the temperature and *B*(*t*) is the baseline determining the amount of heat flow. The reaction progress at the temperature related to time (heating rate) is calculated using the summation of the released energy divided by the total released energy^40^

Laminac decomposition initiates (the reaction progress >0.05) at a higher temperature when increasing the heating rate. (Table 1) due to the relaxation time^41^. To investigate the reaction rate as a function of the temperature by varying the heating rate, we converted DSC data using AKTS. The relevant equation is expressed below^40^.

3 $$\frac{d\alpha }{dt}=\frac{(S(t)-B(t))}{{\int }_{{t}_{o}}^{{t}_{end}}(S(t)-B(t))\,dt},$$

As shown in Fig. 3c and Table 1, the reaction rate increases when the heating rate is increased. Excess heat due to a high heating rate is imposed on the samples, and thus the initiation of laminac decomposition occurs at a relatively high temperature kinetically.

**Table 1:** Initiation temperatures and peak reaction rates of laminac decomposition at various heating rates.

Heating rate (°C/min)	Initiation Temp. (°C)	Peak reaction rate (10^−3^-/s)
	Experiment/Simulation	
1	351/349	0.61/0.63
2	360/362	1.5/1.4
4	373/377	3.1/3.0
8	393/391	7.4/7.0

Data are converted and simulated from DSC signals using the AKTS program.

In the Arrhenius theory, the temperature exponentially increases the rate constant or reaction rate^42^. Ultimately, we are able to predict the reaction initiation time by utilizing Arrhenius theory to confirm the long-term stability of BKNO_3_. The equation is expressed below through the respective α vs. *t* reaction profiles using integration.

4 $${t}_{\alpha }={\int }_{o}^{t}dt={\int }_{{\alpha }_{o}}^{\alpha }\frac{d\alpha }{A^{\prime} (\alpha )\cdot \exp \,(-\,\frac{E(\alpha )}{RT(t)})},$$

The simulated values are shown in Fig. 3d and Table 2, representing that the long-period reaction progress with time is dependent on the storage temperature. Although an increase in the storage temperature generally leads to earlier initiation of the decomposition of laminac, it will not start at 120 °C over 800 years, which is the time scale limitation of the AKTS program. In conclusion, the aging of BKNO_3_ due to the deformation of laminac will require infinite time at ambient temperatures. Thus, the aging probably does not originate from internal factors but from external factors such as the humidity level^13^.

**Table 2:** Initiation time according to the storage temperature.

Storage temperature (°C)	Initiation time
120	Over 800 years
160	18 years
200	5 months
240	1 week
280	1 day

Data are simulated from DSC signals using the AKTS program.

### Aging mechanism analysis under accelerated aging

**Figure 4:**
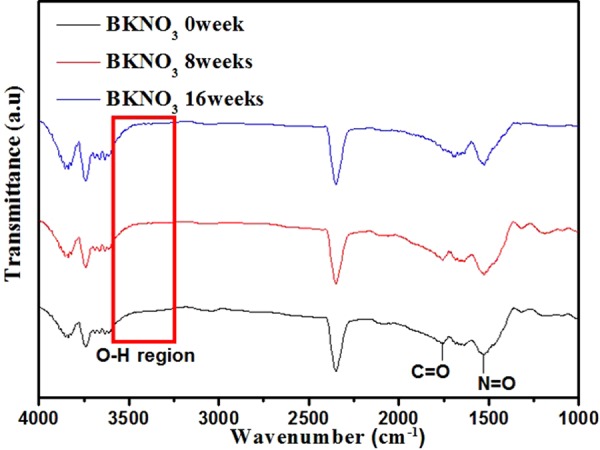
FTIR-spectra of BKNO_3_ under accelerated aging. The hydroxyl group could not be observed, which demonstrates that there is no hydrolysis of laminac

Humidity is a crucial external factor for boron oxidization, resulting in undesirable pre-reactions and decreasing the efficiency of BKNO_3_, despite the fact that only the surfaces of boron particles are oxidized^10^. First, laminac degradation due to humidity was tested using IR spectroscopy under accelerated aging at 71 °C with 50% RH. KNO_3_ can react with water vapor, yielding HNO_3_, a highly acidic material (pKa = −1.0)^43^. Laminac possibly leads to the hydrolysis of ester bonds in laminac. The resulting hydroxyl group and carboxylic acid groups are evidence of the degradation of laminac. As shown in Fig. 4, the C=O stretching band around 1700 cm^−1^ is attributed to the existence of ester bonds in laminac. The N=O stretching band of KNO_3_ was also detected at around 1500 cm^−1^. The bands around 4000–3600 cm^−1^ and 2350 cm^−1^ originate from carbon dioxide in the atmosphere during the experiments^44^. Here, it is noted that there is no band for O-H stretching at around 3400 cm^−1^, suggesting that humidity does not affect laminac degradation^45^.

Accelerated aging samples were characterized to confirm boron oxidization caused by humidity. The relative heats released by thermodynamic aging and reaction rates for kinetic aging were obtained. In addition, the B-O bond and boron oxide shells on the surface were identified using XPS and TEM–EDS, respectively^10^.

**Figure 5:**
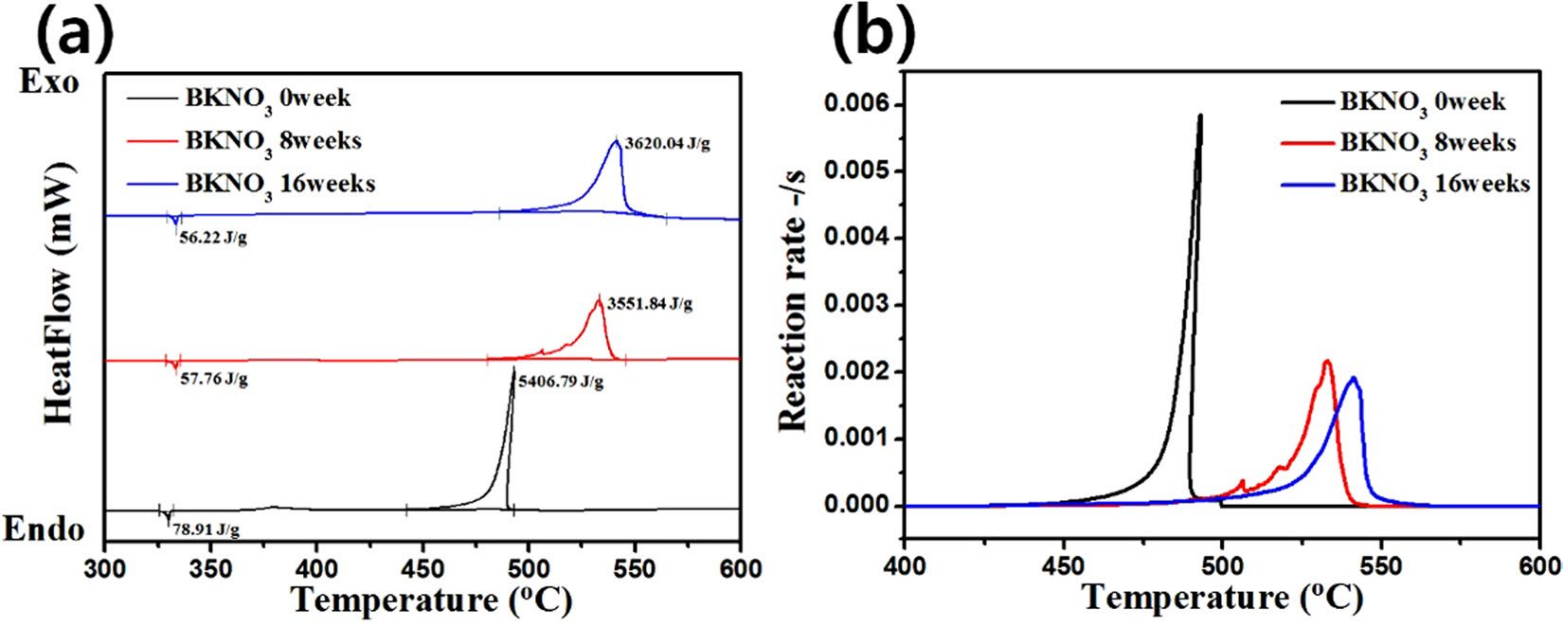
(**a**) DSC profiles and (**b**) reaction rates of BKNO_3_ under accelerated aging. The relative heat of explosion and reaction rate gradually decreased along with extension of humidity aging periods (8 and 16 weeks).

DSC experiments were conducted on the BKNO_3_ samples under accelerated aging at 71 °C with 50% RH for 8 and 16 weeks. It showed that exothermic reactions occur at an onset temperature of around 530 °C (Fig. 5a). The relative heats of reaction, wherein the heat of reaction is divided by the melting heat of KNO_3_, are listed in Table 3. The melting heats of KNO_3_ were obtained from the DSC profiles of pristine, 8, and 16 week samples as 78.91, 57.76 and 56.22 J/g, with heats of reaction of 5406.79, 3551.84, and 3620.04 J/g, respectively. To compensate for the error attributed to highly energetic materials such as explosives, relative values were introduced. The relative heats released were 68.51, 61.53, and 56.44 for the pristine, 8, and 16 weeks-aged boron particles, respectively. This value decreases by up to 18% when compared with that of the pristine sample. In contrast, the samples without induced humidity exhibited rather constant values even after 12 months of aging (Supplementary Fig. S2a and Table S1). It is thus concluded that thermodynamic aging is obviously induced by humidity and progresses gradually over time. Moreover, the reaction rates, which are converted from DSC signals using the AKTS program, are plotted in Fig. 5b and are listed in Table 3. Peak reaction rates of 5.86 × 10^−3^ s^−1^, 2.17 × 10^−3^ s^−1^, and 1.93 × 10^−3^ s^−1^ were calculated for the pristine, 8, and 16 week BKNO_3_ samples, respectively. The reaction rate decreases by 67% over 16 weeks; in other words, kinetic aging occurs. This can be attributed to oxide formation on the boron surface, which plays a role as a barrier to heat propagation, reducing the contact area between boron and KNO_3_, and finally increasing the activation energy for combustion^46^. In contrast, a difference in the reaction rates was not found for no induced humidity (Supplementary Fig. S2b and Table S1), suggesting that kinetic aging also originates from humidity.

**Table 3:** The relative heats, peak reaction rates, and thickness of oxide shells of BKNO_3_ under accelerated aging.

Aging week	Relative ΔH_released_	Peak reaction rate (10^−3^-/s)	Thickness of oxide shells (nm)
0	68.51	5.86	0
8	61.53	2.17	26
16	56.44	1.93	39

Relative heats released ( = reaction heat/melting heat of KNO_3_) and reaction rates are converted from DSC signals using the AKTS program.

**Figure 6:**
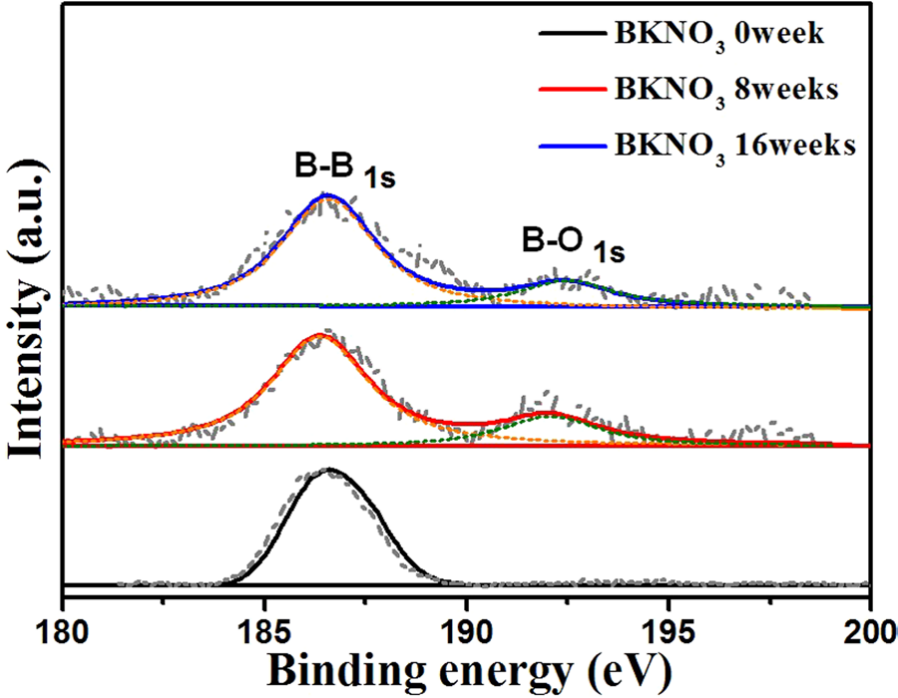
XPS spectra of BKNO_3_ under accelerated aging. In aging samples of 8 and 16 weeks, B-O 1 s peak was observed. It is an evidence of formation of oxide shells on the boron surface.

XPS measurements of the BKNO_3_ samples under accelerated aging are shown in Fig. 6. The B 1 s core electron peak (186.6 eV) is clearly distinguishable from its oxidization state at around 191.5 eV^10,33^. The strongest signal centered at 186.6 eV is typically assigned to the B-B bond of elemental boron. The weak signal at 191.5 eV is due to the B-O bonds in boron oxides. The pristine BKNO_3_ exhibited only the B-B bond signal. In contrast, the B-O bond was detected in both 8 and 16 week samples. This is obviously caused by oxidization between boron and water vapor (humidity). The experiments without induced humidity are good counter evidences for the effect of humidity on the oxidation, wherein both samples stored for 6 and 12 months without induced humidity displayed the B-B bond signals only at 186.6 eV (Supplementary Fig. S3).

**Figure 7:**
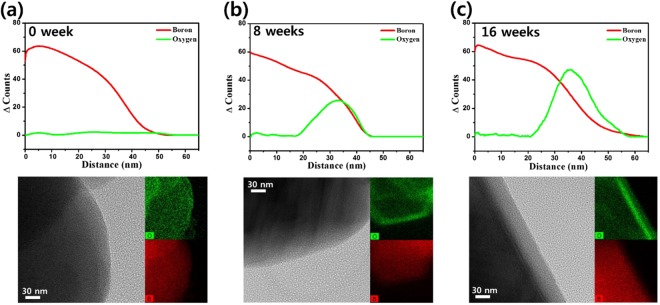
TEM–EDS characterizations of BKNO_3_s under accelerated aging. The thickness of oxide shells gradually increased along with the extension of humidity aging. It corresponds to the tendency of thermal analysis such as relative heat and reaction rate.

The formation of oxide shells is visualized using TEM–EDS in Fig. 7 and Table 3^47^. In the TEM–EDS images, the edges of boron particles under accelerated aging have high counts of oxygen, whereas no oxygen was detected in the pristine sample. Furthermore, the thicknesses of oxide shells gradually increased with time, up to 26 nm and 39 nm for 8 and 16 week samples, respectively. Likewise, oxide shells did not develop for the samples stored at 71 °C without induced humidity for 12 months (Supplementary Fig. S4). That is, both kinetic and thermodynamic aging are induced by humidity levels.

## Discussion

We intensively investigated the aging mechanism of a well-known propellant for PMDs (BKNO_3_). This is essential to secure the sustainable performance of PMDs. The origin of aging was divided into internal factors such as spontaneous pre-reactions and external factors such as humidity levels. First, AKTS simulations combined with thermal analyses confirmed that the decomposition of laminac (a polymer binder) is the fastest exothermic reaction of BKNO_3_, but will not initiate over 800 years even at a storage temperature of 120 °C. The effects of internal factors were ruled out in this way. As for the humidity level (the main external factor), BKNO_3_ samples under accelerated aging at 71 °C in 50% RH were characterized. The heat of reaction and reaction rate, which are related to thermodynamic and kinetic aging, respectively, decreased gradually over time; the heat of reaction decreased by 18% over 16 weeks, whereas the reaction rate decreased by 67%. XPS analysis exhibited a B-O peak (around 191.5 eV) due to boron oxidization. The resulting surface oxide shells were identified through TEM–EDS studies. The thicknesses of oxide shells gradually increased up to 39 nm over 16 weeks and were reciprocally related to the heat of reaction and the reaction rate. In conclusion, the primary origin of BKNO_3_ aging is the high humidity condition present during the incorporation process of propellants into PMDs to prevent undesirable explosions. The high humidity level leads to the formation of oxide shells on the boron surface and their presence lowers the potential energy (explosive energy) of the system. The reaction rate is also reduced because the oxide shells play a role as a barrier to heat propagation and the contact between boron and KNO_3_.

## Methods

### Materials

Potassium nitrate particles of around diameter of 6 µm were purchased from Hummel Croton. Highly pure amorphous boron powder with a particle size of around diameter of 1 µm was prepared by Rockwood Lithium, and the polymer binder (LAMINAC 4116) was purchased from Ashland.

### Accelerated aging method

The BKNO_3_ propellants were stored in a furnace that sustained the temperature at 71 °C. The humidity level was then controlled by storing the samples in a 4.7 L airtight container. Relative humidity (RH) was calculated using the ideal gas law and water vapor pressure at 71 °C. For example, 0.47 g of purified water was incorporated in the airtight container to produce a RH of 50%. The calculation procedure for 50% RH is shown in Eqns (2) and (3).

The mole of water (50% RH 4.7L 71 °C)

5 $$=\,\frac{0.154\,atm\times 4.7L}{0.082\,atm\cdot L\cdot \,{K}^{-1}\cdot mo{l}^{-1}\times 344\,K}=0.0259\,mol,$$

The amount of water (50% RH 4.7L 71 °C)

6 $$=\,0.0259\,mol\times 18\,g\cdot mo{l}^{-1}=0.47\,g,$$

### TGA analysis

Investigations using Q500 V50 were conducted while heating the samples from 30 to 550 °C at a rate of 10 °C/min under a nitrogen atmosphere. The weight of the BKNO_3_ sample for each measurement was about 10 mg and thermogravimetric signals were recorded.

### DSC measurements & AKTS simulation

DSC was performed using a DSC 4000 (PerkinElmer). DSC data for AKTS simulations were obtained at various heating rates (1, 2, 4 and 8 °C/min.). In addition, the measurements of the heats of reaction were conducted while heating the samples to 600 °C at a scanning rate of 2 °C/min. A slow heating rate could generate thermodynamic information with a sufficient relaxation time. Kinetic analysis of the thermally simulated processes with AKTS used the DSC data with various heating rates.

### IR spectroscopy

Fourier transform infrared spectra were obtained using a Cary 600 spectrometer equipped with a MCT-A (mercury cadmium telluride) detector with 5 mg samples in a powdered state.

### XPS characterizations

The XPS spectra were obtained using a MultiLab 2000 with monochromated Al Kα X-rays and a hemispherical analyzer with a pass energy of 30 eV. The BKNO_3_ samples were prepared on conductive copper tape. Background subtraction was performed using a Shirley background and MonoXPS.

### TEM–EDS images

TEM images, elemental mapping, and EDS were performed using a Titan G2 ChemiSTEM Cs Probe (FEI Company, Netherlands) with an acceleration voltage of 200 kV. The samples for the TEM observations were prepared by immersing a TEM mesh into a boron particle suspension in ethanol. After being dried in air, the TEM mesh was then characterized.

## Electronic supplementary material

Supplementary Information
