# Supplementary material for: Study on the Aging Mechanism of Boron Potassium Nitrate (BKNO3) for Sustainable Efficiency in Pyrotechnic Mechanical Devices
Source: Sci Rep. 2018 Aug 6;8:11745. doi: 10.1038/s41598-018-29412-8 (PMC6078969; doi:10.1038/s41598-018-29412-8)
Supplement: Supplementary file 1 — Supplementary Information [file 41598_2018_29412_MOESM1_ESM.doc]

Electronic Supplementary Information

*For*

Study on the Aging Mechanism of Boron Potassium Nitrate (BKNO_3_) for Sustainable Efficiency in Pyrotechnic Mechanical Devices

Junwoo Lee,^1^ Taewan Kim,^1^ Seung Un Ryu,^1^ Kyoungwon Choi,^1^ Gil Hwan Ahn,^2^ Jong Gyu Paik,^3^ Byungtae Ryu,^3^ Taiho Park,*^1^ and Yong Sun Won*^4^

*1. Chemical Engineering, Pohang University of Science and Technology (POSTECH), 77 Cheongam-Ro, Nam-gu, Pohang, Kyoungbuk, Korea.*

*2. Hanwha Corporation Defense R&D Center, Daejeon 34068, Korea*

*3. Agency for Defense Development, Daejeon 305-152, Korea*

*4. Chemical Engineering, Pukyong National University,* 365 Sinseon-Ro, Nam-gu, Busan 48547*, Busan, Korea.*

* Corresponding author:

[*taihopark@postech.ac.kr*](mailto:taihopark@postech.ac.kr)

[*yswon@pknu.ac.kr*](mailto:yswon@pknu.ac.kr)

**
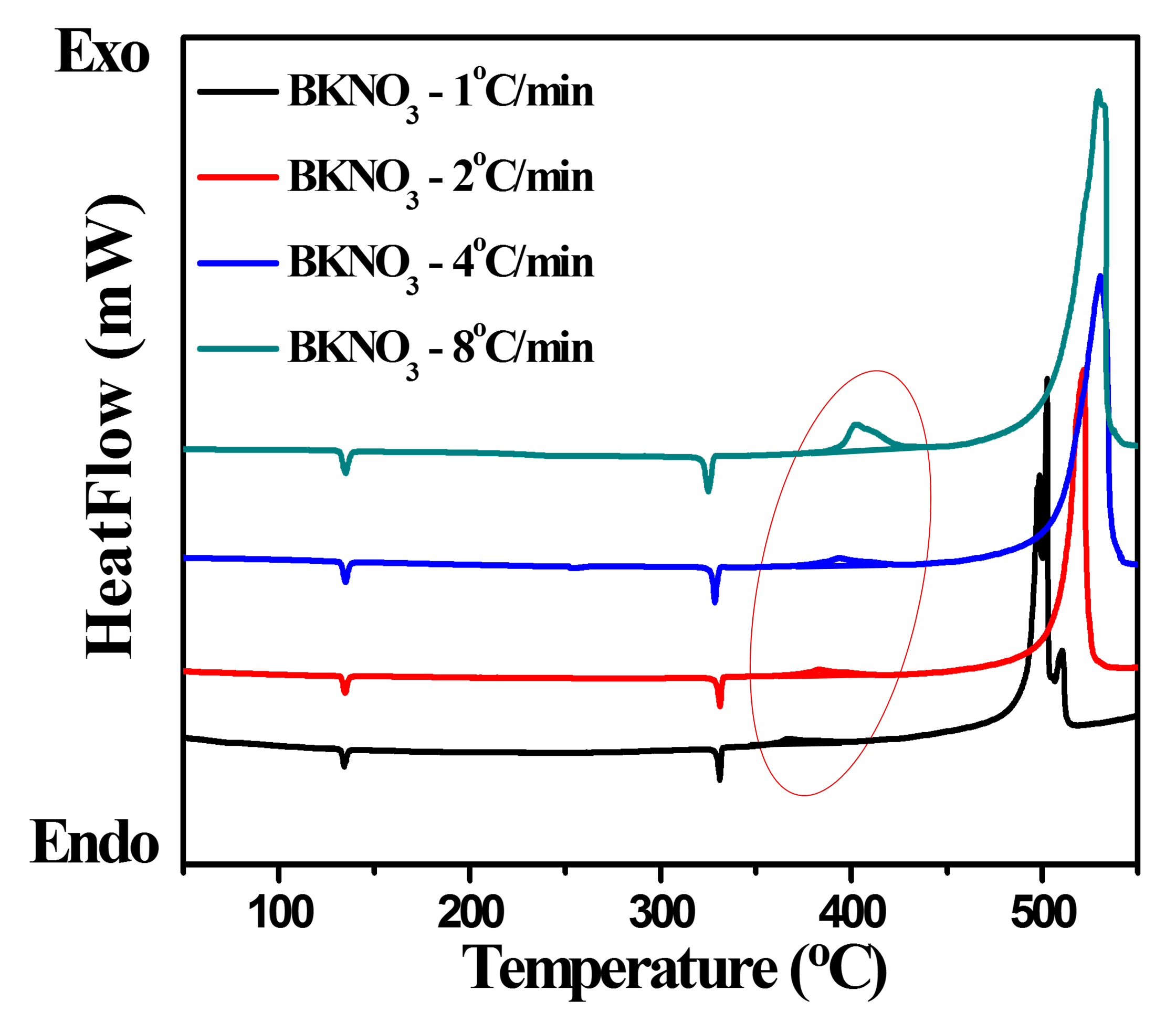
**

**Figure S1.** The DSC profiles of BKNO_3_ (1, 2, 4, 8 ^o^C/min)


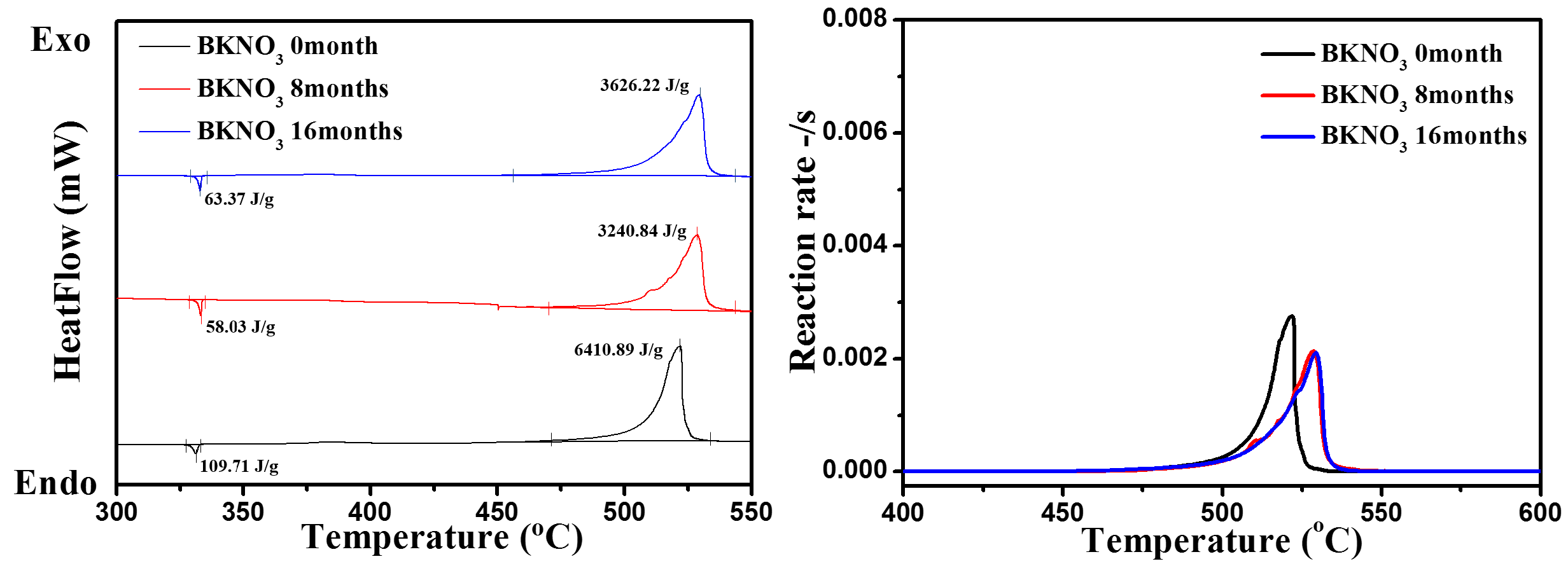


**Figure S2.** (a) DSC profiles and (b) reaction rates of the accelerated aging BKNO_3_s without humidity

**Table S1. The relative heats and peak reaction rates of the accelerated aging BKNO_3_s without humidity**

| Aging month | Relative ΔH_released_ | Peak reaction rate (10^-3^ -/s) |
| --- | --- | --- |
| 0 | 58.46 | 2.76 |
| 6 | 56.38 | 2.14 |
| 12 | 57.23 | 2.09 |


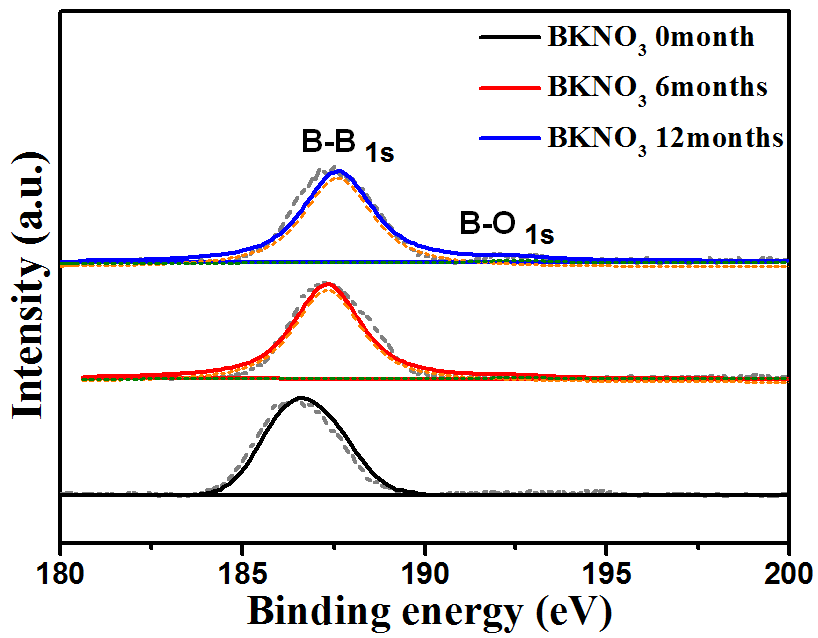


**Figure S3.** XPS spectrums of the accelerated aging BKNO_3_s without humidity


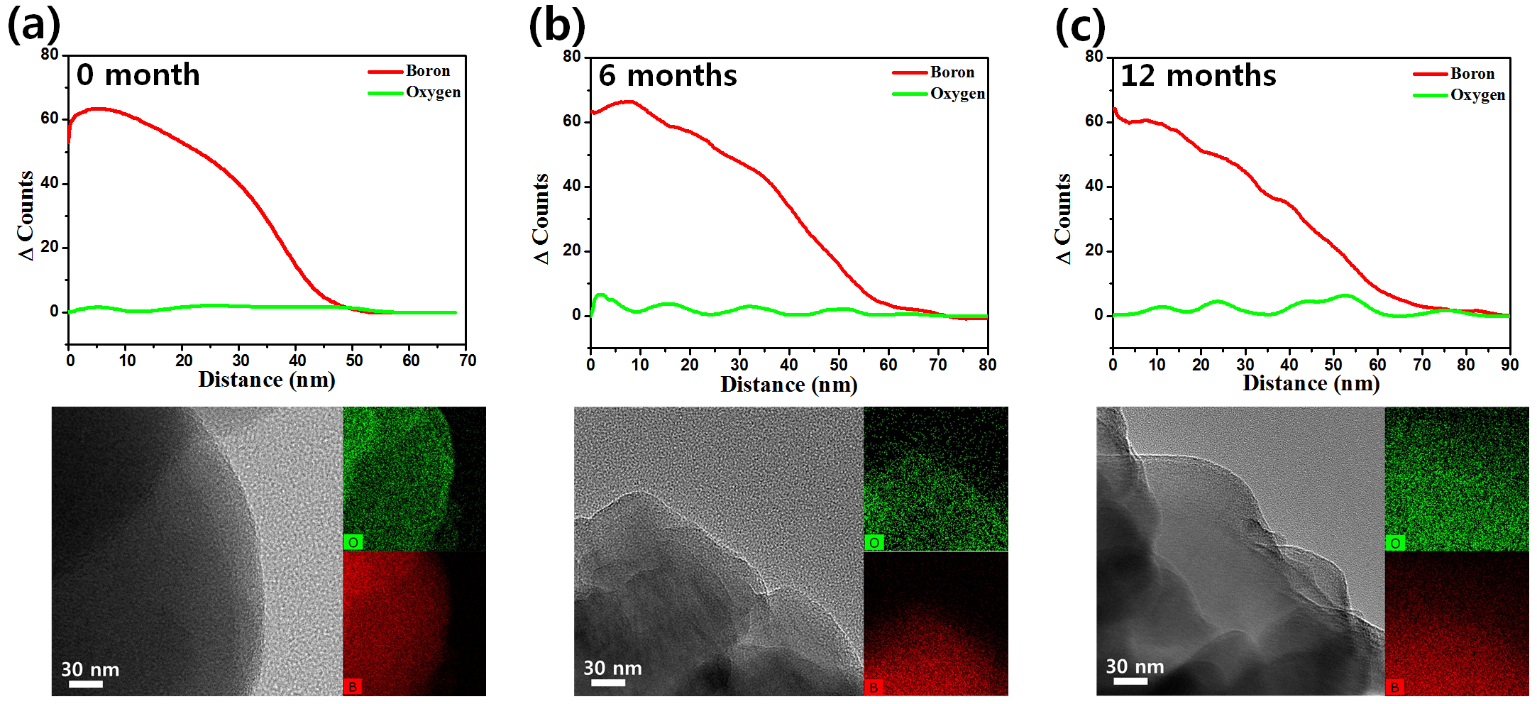


**Figure S4**. TEM – EDS characterizations of the accelerated aging BKNO_3_s without humidity
